# Assessment of the potential neuroprotective effect of PhKv toxin, isolated from *Phoneutria nigriventer* venom, in vesicular acetylcholine transporter‐deficient mice

**DOI:** 10.1002/alz.091549

**Published:** 2025-01-09

**Authors:** Ana Caroline Nogueira‐Souza, Cristina Guatimosim Fonseca, Hèlia Tenza‐Ferrer, Marcia Helena Borges, Luiz Armando Cunha de Marco, Marco Aurélio Romano‐Silva, Marcus Vinícius Gomez

**Affiliations:** ^1^ Federal University of Minas Gerais, Belo Horizonte, Minas Gerais Brazil; ^2^ Ezequiel Dias Foundation, Belo Horizonte, Minas Gerais Brazil; ^3^ Santa Casa BH Teaching and Research Institute, Belo Horizonte, Minas Gerais Brazil

## Abstract

**Background:**

Acetylcholine, a neurotransmitter critical for cognitive functions, including attention, memory, and sociability, is essential for maintaining synaptic integrity. Deficits in acetylcholine levels are linked to cognitive impairments. Heterozygous VAChT KD (VAChT KDHET) mice, characterized by reduced vesicular acetylcholine transporter protein production, exhibit cognitive impairments due to diminished acetylcholine release. This study explores the therapeutic potential of PhKv toxin, derived from the Brazilian wandering spider *Phoneutria nigriventer*, as an acetylcholinesterase inhibitor, aiming to prolong acetylcholine presence in the synaptic cleft of murine subjects.

**Methods:**

Cognitive deficits in VAChT KDHET mice (n = 8) were assessed using the novel object recognition task. Thirty‐six VAChT KDHET mice were divided into three groups: PhKv (n = 12), galantamine (n = 12), and control/sham (n = 12). The PhKv group received subcutaneous vehicle (0.9% saline) and intracerebroventricular PhKv toxin (100 pmol/site). The galantamine group received subcutaneous galantamine (1 mg/kg) and intracerebroventricular vehicle (1x PBS). Controls received both vehicles. Preceding treatments, mice underwent habituation (HAB) and familiarization (FAM) in the novel object recognition test. Post‐FAM, mice received subcutaneous galantamine or vehicle, followed by intracerebroventricular PhKv or vehicle to minimize FAM‐related injection effects. After 24 hours, long‐term memory (LTM) was assessed.

**Results:**

VAChT KDHET mice displayed impaired object recognition memory, with sex‐related short‐term memory differences. PhKv‐treated mice exhibited memory improvements comparable to galantamine‐ or vehicle‐treated mice, indicating potential memory enhancement. Further analysis revealed memory improvement in both vehicle‐treated and naive mutant mice.

**Conclusion:**

PhKv shows promise in memory improvement, warranting in‐depth investigations into its mechanisms. Ongoing research explores the molecular aspects of PhKv’s action on acetylcholinesterase. Our study suggests PhKv’s therapeutic relevance in addressing cognitive deficits associated with acetylcholine dysregulation.

